# Long-term Functional Outcome and Satisfaction After Surgical Management of Cuboid Fractures

**DOI:** 10.1177/24730114251388656

**Published:** 2025-11-17

**Authors:** Esmee W.M. Engelmann, Jens A. Halm, Tim Schepers

**Affiliations:** 1 Amsterdam UMC–Location AMC, the Netherlands

**Keywords:** cuboid, foot trauma, ORIF, Chopart, Lisfranc, functional outcome

## Abstract

**Background:**

Cuboid fractures are rare, and there is a scarcity in evidence of the long-term functional outcome of these fractures in literature. This study aimed to evaluate functional outcome, complications, quality of life, and patient satisfaction after surgical treatment of cuboid fractures.

**Methods:**

This retrospective study was conducted at a level 1 trauma center, reviewing patients ≥16 years with cuboid fractures operated between 2014 and 2024. Functional outcome was assessed using the Foot Function Index (FFI), the American Orthopaedic Foot & Ankle Society (AOFAS) midfoot score, and EQ-5D quality of life questionnaire. Complications such as nonunion, infection, and secondary arthrodesis were recorded.

**Results:**

The total cohort of surgically managed patients was 45 (28 females and 17 males), with a median age of 39.1 (IQR, 24.2) years and median follow-up of 67.0 (IQR, 91) months. A subset of 42 patients were treated with open reduction and internal fixation within 4 weeks after the injury. Overall, AOFAS was fair to good (median 76, range 34-100) and treatment satisfaction was high (mean 8.7/10, SD 1.1). There were no infections and no cases of nonunion. Secondary calcaneocuboid joint fusion was required in only 2 patients. In multivariate linear regression analysis, the type of fixation (cuboid plate) was significantly associated with better AOFAS (*P* = .03, *R*^2^ 0.11).

**Conclusion:**

Surgical intervention aimed at restoring articular congruence and column length with cuboid plate fixation can lead to union with overall fair to good functional outcome and high patient satisfaction.

**Level of Evidence:**

Level III, retrospective cohort study.

## Introduction

With an annual incidence of only 1.8/100 000 fractures, cuboid fractures are considered rare.^1,2^ This is largely due to the bone anatomy and protected location of the cuboid in the midfoot.^3^ The cuboid is a crucial component of the lateral column of the foot and is involved in all intrinsic movement.^4^ It articulates with the calcaneus (proximal facet), lateral cuneiform (medial facet), and fourth and fifth metatarsals (distal facets).^5^ The cuboideonavicular joint facilitates rotational and gliding movement, whereas movement in the calcaneocuboid joint (CCJ) is very limited. In fact, an in vitro model demonstrated that all significant lateral column movement occurs distally at the tarsometatarsal joints (TMTJs).^6^

The majority of cuboid fractures are isolated avulsion fractures.^7^ A smaller yet more severe group includes lateral compression fractures that are rarely isolated but usually associated with other midfoot injuries including Chopart or Lisfranc injuries.^8,9^ Hermel and Gershon-Cohen were the first in 1953 who coined these injuries to the cuboid “nutcracker fracture,” with the nutcracker being the calcaneus and the fourth and fifth metatarsals.^2^ Treatment of cuboid fractures depends on the injury characteristics. Nondisplaced and avulsion fractures may be managed nonoperatively through immobilization and a period of nonweightbearing.^3,10^ Surgical indications include loss of lateral column length (>2 mm) and articular incongruency (>1 mm displacement).^3,7,11^ A number of different surgical methods have been proposed, including open reduction and internal fixation (ORIF) with plates and screws and external fixation. In the available literature, surgical management is suggested to avoid adverse biomechanical and functional consequences as well as arthritis, stiffness, and chronic pain.^12^ However, there is limited evidence in the literature regarding the long-term functional outcome of these fractures.^10^ Therefore, we aimed to provide evidence on long-term functional outcome after operative management of cuboid fractures.

## Methods

### Study Design and Patients

A retrospective cohort of patients with cuboid fractures who were treated surgically at a level 1 trauma center between January 1, 2014, and January 1, 2024, was analyzed. Institutional review board approval and written informed consent were obtained.

### Patient Selection

All patients who were surgically treated for cuboid fractures were selected. Patients below the age of 16 years on the day of injury, patients with foot compartment syndrome, open fractures, or injuries older than 4 weeks were excluded. All patients had at least 1 year of follow-up. Included patients were followed by clinical evaluation at the outpatient clinic and were sent a questionnaire between 2021 and 2024, at least 1 year after surgery. Loss to follow-up was recorded.

### Operative Technique

For each patient, a treatment strategy was chosen by one of 3 foot ankle surgeons (trauma surgeons, foot and ankle fellowship trained, each with more than 10 years of independent practice). In general, ORIF was indicated in case of an intra-articular fracture with displacement of more than 1 mm at the articular surface or more than 3 mm of column length loss, in a patient fit for surgery.^10^ Patients were positioned supine with a bump under the ipsilateral thigh to invert the foot. A dorsolateral approach was used, centered over the cuboid along the fourth metatarsal axis and proximally to a point about 5 mm below the tip of the fibula. The length of the incision was dictated by the extent of the injury, whether or not both or a single joint was involved. Dorsolateral sural nerve branches may be identified and protected. The extensor digitorum brevis muscle was retracted dorsomedially and the peroneal muscles in the plantar direction. A small distraction device was commonly used. Subsequently, the lateral cortex, which usually shows signs of blow-out, can be lifted dorsomedially. The impacted joint surface was debrided and deimpacted with a periosteal elevator. If this joint surface is fractured, several 1.0-mm Kirschner wires were used to support this thin shell. Either a local graft or a graft from a small window of the distal medial tibia was inserted. Occasionally, a demineralized graft was used from the bone bank. After careful radiographic control, the lid was closed and either a 2.0- or a 2.4- mm straight plate was used as rim plate to support a single joint injury, or an anatomical 2.7-mm plate was used to support both joints. Most screws used were locking screws to create a fixed-angle construct to prevent collapse. The wound was closed after rinsing. The standard postoperative instruction was nonweightbearing for 6 weeks in a cast.

### Variables

Patient-related clinical and radiographic data were extracted from the electronic hospital database. Variables included were age at injury, gender, body mass index, tobacco use, mechanism of trauma, Injury Severity Score, injury pattern, classification, and concomitant ipsi- or contralateral lower extremity injuries.^13^ Fractures were classified according to the AO/Orthopaedic Trauma Association (AO/OTA) classification, classification by Fenton et al,^14,15^ and a simplified classification for this study. Based on the AO/OTA and Fenton classifications, we divided the surgically treated cuboids into type 1 fractures (proximal at the CCJ, Chopart-like), type 2 fractures (distal at the TMTJ, Lisfranc-like), and type 3 fractures (combined).

Data were collected on the type of treatment, follow-up, complications, functional outcome, quality of life, and treatment satisfaction. Complications were defined as delayed, mal- or nonunion, infection, and secondary arthrodesis (SA). Functional outcome was quantified using the Foot Function Index (FFI, best score 0 points), and the American Orthopaedic Foot & Ankle Society midfoot score (AOFAS, best score 100 points). The AOFAS score was divided into groups according to the literature: a score of 90 to 100 was graded as an excellent result; 75 to 89 as good; 50 to 74 as fair, and less than 50 points was graded as a failure or poor outcome. Quality of life was measured by EuroQol, Five Dimensions (EQ-5D). Assessment of perceived general health was done using a visual analog scale (VAS) score ranging from 0 to 10, in which 10 represented excellent general health. Treatment satisfaction was also measured using the VAS of 0 to 10, in which 10 represents the best possible satisfaction. Work status and level of activity were rated based on a modification by Arntz et al,^16^ including pretrauma activity and work, some change in level or with limitations, and did not resume activities and work.

### Statistical Analysis

The statistical analysis was performed using the Statistical Package for the Social Sciences, version 29 (IBM Corp, Armonk, NY). Numeric data are expressed with means with SD or median with IQR. Categorical data are shown as numbers with percentages. Independent sample *t* tests and analysis of variance with a significance level of .05 were used to compare means. Additionally, a multivariate linear regression was performed to identify independent predictors of functional outcome. Variables included were classification, joint dislocation, concomitant injuries, temporary fix and type of fixation, implant removal, and secondary arthrodesis. A manual backward linear regression was performed. Variables were included into the multiple regression model if *P* < .05.

## Results

### Demographics

**Figure 1.**
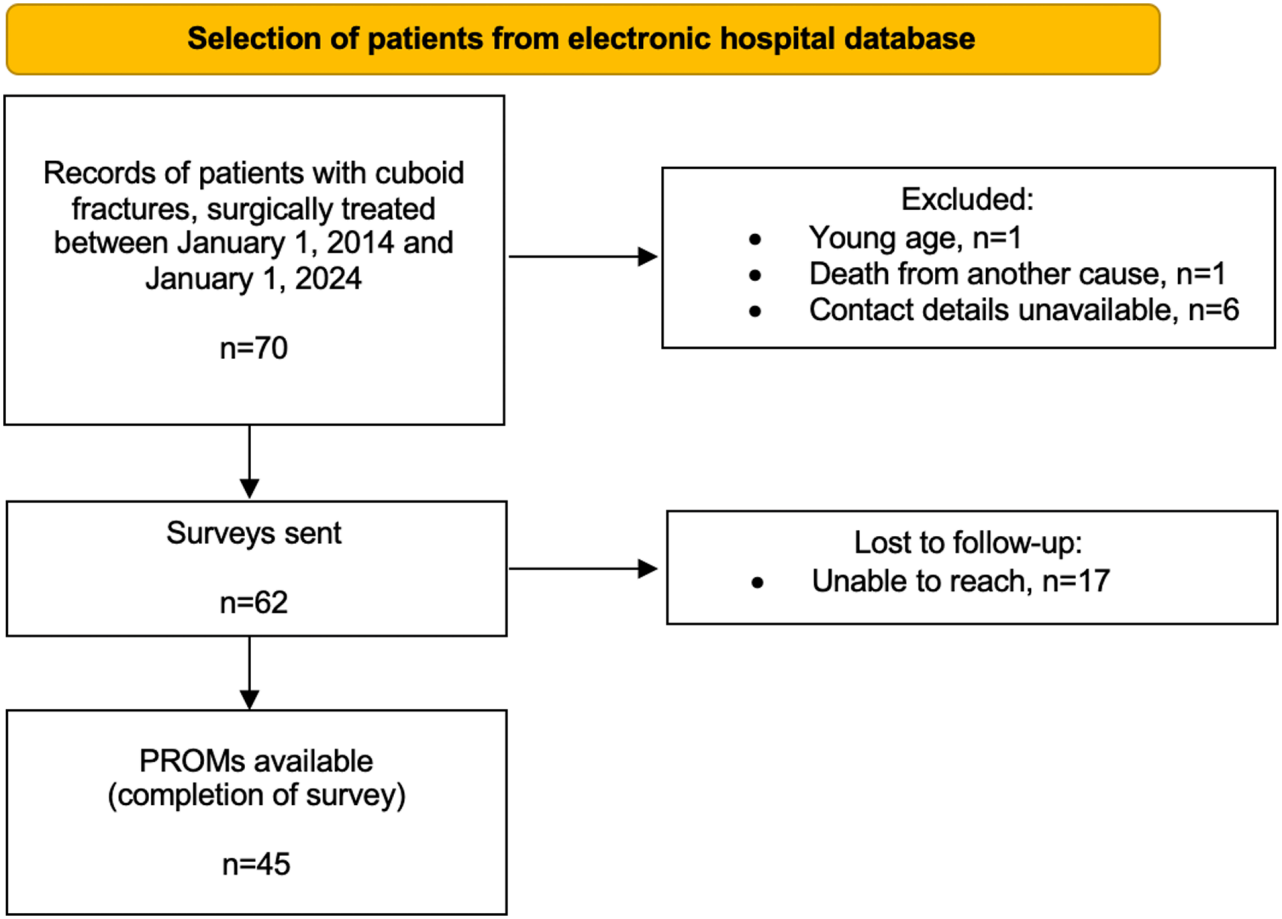
Patient selection process.

The total number of patients included in this study is 45. Seventy patients with cuboid fractures who were treated surgically at our level 1 trauma center were identified. Eight patients were excluded per the predetermined exclusion criteria. Questionnaires were sent to the remaining 62 patients, of whom 45 responded (72.6%; Figure 1). There were 28 males and 17 females, with an overall median age at the day of trauma of 39.1 (IQR, 24.2) years. Median body mass index was 24.1 (IQR, 5.8) and 17.8% of patients reported tobacco use (n = 8/45). The majority of patients (n = 35/45, 77.8%) were referred from other hospitals, of which 32 patients were referred within 4 weeks after trauma (median, 9 days; IQR, 12) and 3 patients were referred in a later stage (median, 6 months; IQR, 4). Median follow-up time from the day of injury was 67 (IQR, 91) months.

### Fracture Classification and Injury Pattern

The mechanisms of trauma were fall or inversion during daily activities (n = 19/45), fall from height (n = 11/45), motor vehicle accident (n = 11/45) and crush (n = 4/45). Median Injury Severity Score was 4 (range, 4-34; IQR, 0). All patients were diagnosed using both conventional radiography and computed tomographic imaging. With respect to the AO/OTA classification, 80% of patients (n = 37/45) sustained an intra-articular fracture injury (84 C-b; cuboid [84] complete articular fracture [C], multifragmentary [b]), and 7 patients had a severe impaction injury that could not be classified using this system.^14^ In terms of the classification by Fenton et al,^15^ 62.2% of patients (n = 28/45) had a type 5 fracture, defined as cuboid fractures with associated midtarsal disruption. This type was subdivided into 5a (n = 8/28), with disruption and shortening of the lateral column, and 5b (n = 20/28), with disruption of the medial (navicular fracture) and lateral columns. A total of 9 patients had a type 4 fracture, described as a cuboid fracture with an associated intra-articular injury affecting the tarsometatarsal complex (TMTJ 4 and 5). One of these patients, however, did not fit the description, because of associated injuries to the first to third metatarsals instead of fourth and fifth. Next, 7 patients sustained intra-articular fractures of the cuboid involving the TMTJ or CCJ or both; however, only 1 of these was isolated and thus 6 did not fit the description of this classification. The Fenton classification was therefore not found suitable for the study population. Based on our simplified classification, there were 11 patients with type 1 fractures with proximal intra-articular involvement at the CCJ, 19 patients with type 2 fractures with distal (TMT) joint disruption, and 15 patients with type 3 injuries (both proximal and distal).

Four patients had an isolated cuboid fracture (n = 4/45, 8.9%), whereas the majority had additional fractures of the foot including calcaneus fractures (n = 7/45, of which n = 5 anterior process), navicular fractures (n = 21), cuneiform fractures (n = 15), and/or metatarsal fractures (n = 22). Of the 41 patients with other ipsilateral foot injuries, 36 (87.8%) required surgery for those injuries. This included ORIF of the navicular (n = 17), the Lisfranc joint complex (n = 10), calcaneus (n = 5), and cuneiform bone (n = 4). Three patients had PA of the TMT 1-2 and 1 patient of the talonavicular joint. Seven patients (n = 7/45, 15.6%) presented with a fracture-dislocation of the calcaneocuboid joint (n = 3), tarsometatarsal joint (n = 3), or both (n = 1). One patient had a Gustilo-Anderson grade 1 open fracture. None of the included patients sustained fractures to the contralateral foot or ankle.

### Management

**Figure 2.**
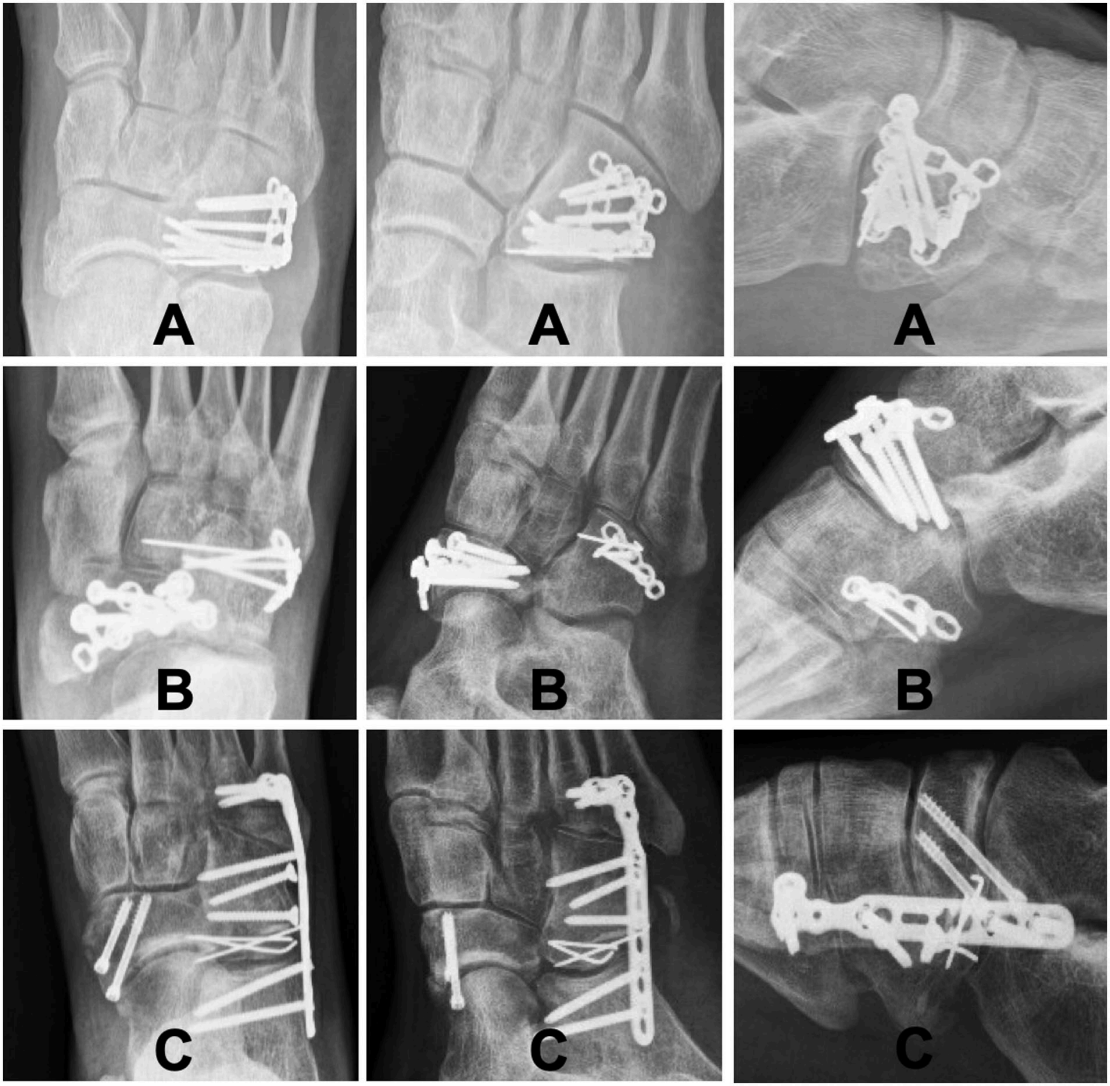
Examples of fixation techniques used. Left to right: anteroposterior, oblique, and lateral radiographs. Top to bottom: (A) ORIF cuboid with VA locking cuboid plate 2.4/2.7, (B) ORIF cuboid with 2.0 plate and 1.0 K-wire for additional fixation of small fragment, ipsilateral navicular fracture, (C) ORIF cuboid with locking bridge-plate (calcaneus, cuboid, metatarsals 4 and 5), 2x 2.7 cortical screws, 1.0 K-wires, as part of Chopart fracture-dislocation including navicular fracture.

Before definitive surgery, 4 patients (n = 4/45, 8.9%) were temporarily treated with either external fixation of the foot (medial and lateral columns, n = 3) or percutaneous Kirschner wire (K-wire) fixation (n = 1). A total of 42 patients were surgically managed with ORIF within 4 weeks after the injury (median, 12.5 days; IQR, 10.8). The fixation was achieved by using a cuboid plate (n = 20, of which 2 with temporary K-wire fixation of the CCJ, 1 with CCJ suture, and 2 with autologous bone graft), joint-spanning bridge plate (n = 14, of which 2 with autologous bone graft and 1 with bone void filler), K-wires for 8 weeks (n = 2), screws only (n = 1), or a combination of the previous (n = 5). Examples of different fixation techniques are demonstrated in Figure 2. Three patients that were referred late (4, 6, and 8 months after injury) were managed with a correction osteotomy, bridge plate and K-wire fixation, correction osteotomy, and cuboid plate fixation and secondary CCJ arthrodesis, respectively.

### Complications

Union was achieved in all patients. The majority of patients (n = 27/45, 60%) required more than 1 operation, with a median of 2 (IQR, 1). Implants were removed in 53.3% of the patients (n = 24), including all patients after bridge-plating (median, 3.5 months; IQR, 1.5). There were no patients with infection. Secondary calcaneocuboid arthrodesis due to painful osteoarthritis after ORIF was performed in 2 patients (n = 2/42, 4.5%).

### Functional Outcome and Other Patient-Reported Outcomes

Overall AOFAS was fair to good, and treatment satisfaction was high. In patients who were treated surgically within 4 weeks after injury (n = 42), the median AOFAS score was 76 (range 34-100) and the FFI was 20.5 (0-59). The mean EQ-5D score was 7.0 (SD 1.7), mean perceived health score was 7.7 (1.6) and mean treatment satisfaction score was 8.7 (1.1). In patients who were treated later (n = 3), the median AOFAS score was 84 (range 30-90) and the FFI was 7 (3-59). The mean EQ-5D score was 6.0 (SD 1.7), mean perceived health score was 8.9 (0.9), and the mean treatment satisfaction score was 8.7 (0.7).

With the numbers available, no significant differences were detected in outcome scores (functional, quality of life, health and satisfaction) between subgroups in terms of classification, injury type, patients with or without joint dislocation, and type of fixation (Table 1). In multivariate linear regression analysis, the type of fixation (cuboid plate) was significantly associated with better AOFAS (*P* = .03, *R*^2^ = 0.11). The presence of concomitant foot fractures (*P* = .02) negatively impacted FFI (*R*^2^ = 0.12).

**Table 1.** Comparison of Means per Subgroup (Initial Treatment).^a^

Subgroup (n = 45)	AOFAS	FFI
Novel classification (n)		
Type 1 (11)	77.7 (17.6)	16.6 (13.1)
Type 2 (19)	75.0 (16.3)	21.3 (20.6)
Type 3 (15)	69.5 (20.4)	28.8 (17.3)
*P*	.5	.2
Fenton classification (n)		
Type 1 (1)	88	3
Type 3 (7)	75.9 (16.0)	18.7 (10.9)
Type 4 (9)	76.3 (13.7)	19.4 (18.7)
Type 5a (8)	63.4 (19.6)	30.5 (19.1)
Type 5b (20)	75.4 (19.4)	23.3 (19.8)
*P*	.5	.5
Type of fixation (n)		
Screws only (1)	100	2
K-wires only (2)	67.5 (17.7)	28 (28.3)
Cuboid plate (21)	75.2 (13.6)	21.4 (16.8)
Bridge plate 14)	69.3 (19.4)	27.7 (21.4)
SA (1)	30	59
*P*	.1	.3

Abbreviations: AOFAS, American Orthopaedic Foot & Ankle Society Score; FFI, Foot Function Index.

a Data presented as mean (SD).

In terms of daily activities, it was reported that the majority of patients could walk barefoot (without complaints n = 26/45, 56.5%, or minor complaints n = 19/45, 41.3%). More than half of the patients reported stiffness, limited to morning only (n = 13/45, 28.3%) or continuous (n = 12/45, 26.1%). The majority of patients were able to run more than 600 m (n = 28/45, 60.1%). Overall, 85.4% of patients returned to work (n = 35/41 patients who worked prior to trauma), of which 2 patients had to change the type or intensity of work. In total, 62.5% were able to return to the same level of sports (n = 25/40 who performed sports prior to trauma).

## Discussion

In this cohort, 4 of 5 patients were referred from other hospitals, reflecting the ongoing centralization of complex foot and ankle trauma surgery in the Netherlands. More than 90% of patients had additional ipsilateral foot fractures, underscoring the complexity of these injuries. Patients were primarily managed with ORIF using cuboid plates (2.7 anatomical, 1.5/2.0, or 2.7 bridge). Notably, no primary arthrodeses were performed, and only 2 patients required secondary CCJ fusion. There were no cases of infection or nonunion. Average long-term functional outcome was fair to good, with high levels of patient satisfaction and perceived health.

Since the original comparison of cuboid fractures as a nutcracker injury in 1953, the literature on management and outcomes has remained sparse.^2^ Most studies consist of case reports or small case series with only a few patients.^17-20^ More recently, slightly larger cohorts of more than 10 patients were added to the literature.^12,21^ For example, Weber and Locher^12^ studied the short to midterm results of 12 cuboid compression fractures managed with plate or screws only, reporting an average AOFAS score of 86. Our results also demonstrated low rates of complications and acceptable functional outcome, suggesting that the threshold for surgery should be low. Nevertheless, it should be noted that this is mainly based on retrospective data, which could hamper managing expectations toward patients.

Cuboid fractures rarely occur in isolation (8.9% in this cohort), often presenting as part of more extensive foot injuries such as Chopart, Lisfranc, or combined complex foot injury.^10,22^ This was shown to be negatively associated with functional outcome as measured by Foot Function Index in this study. Current classification systems like AO/OTA and Fenton lack the granularity to capture the diverse fracture patterns observed in this cohort. A simplified classification used in this study demonstrated a near-equal distribution of fractures involving proximal (CCJ), distal (TMT), or both joint complexes. To date, a classification that is validated for clinical practice (ie, guidance for management and prediction of outcome and prognosis) is still lacking.

Surgical management of cuboid fractures remains underexplored, with limited consensus on indications for surgery. However, a step-off of more than 1 mm at the articular surface or column length loss exceeding 3 mm are generally accepted as surgical thresholds.^10,23^ Management of patients is subsequently adapted to the injury pattern, including concomitant fractures. Cuboid fracture surgery should focus on early restoration of articular surfaces and lateral column length.^12^ Regarding the latter, using the contralateral side as a mirror-image may be helpful.^23^ Advanced surgical planning tools, including a 3D guide for fracture reduction, have been proposed, although their clinical utility requires further evaluation.^21^

To our knowledge, no comparative studies between nonoperative and operative management are available in the field of displaced intra-articular cuboid fractures. Herein lies one of the benefits of the current study. It could provide guidance for expected long-term functional recovery (including daily activities such as walking, running, return to work and sports), quality of life, and complication rates, which can improve patient counseling.

### Limitations

Strengths of the current cohort are related to use of patient-reported outcome instruments and long-term follow-up. This study is the largest case series reporting on long-term functional outcome, complications, and perceived health after surgically treated cuboid fractures. Given that the majority of cuboid fractures occurred with other ipsilateral foot injuries, the results cannot be extrapolated. There is also potential risk of selection bias because of the very low incidence of cuboid fractures, and consequently, the retrospective study design with small sample size and lack of control group. Results may also have been affected by the fact that in general, the more severe injuries were referred to our foot and ankle trauma expertise center (referral bias). And 45 of 62 patients (72.6%) completed the questionnaires; thus, nonresponse and referral bias may limit generalizability. In addition, differences between responders and nonresponders were not captured. Future (international) collaboration in multicenter studies may improve research opportunities aimed at evaluating treatment and outcome of all cuboid fractures.

## Conclusion

Cuboid fractures are rare and often occur in the context of complex foot trauma. Because of this setting of multiple concomitant foot injuries, the clinical use of functional outcome results is limited. Early recognition and surgical intervention aimed at restoring articular congruence and column length with plate fixation can lead to union with overall fair to good functional outcome and high patient satisfaction.

## Supplemental Material

sj-pdf-1-fao-10.1177_24730114251388656 – Supplemental material for Long-term Functional Outcome and Satisfaction After Surgical Management of Cuboid Fractures Supplemental material, sj-pdf-1-fao-10.1177_24730114251388656 for Long-term Functional Outcome and Satisfaction After Surgical Management of Cuboid Fractures by Esmee W.M. Engelmann, Jens A. Halm and Tim Schepers in Foot & Ankle Orthopaedics
